# P029 The double edged sword of therapy: lessons from an iatrogenic complication

**DOI:** 10.1093/rap/rkag113.059

**Published:** 2026-11-03

**Authors:** Jasiththa Anpalagan

**Affiliations:** Queen Elizabeth Hospital, King’s Lynn, United Kingdom

## Abstract

**Introduction:**

Giant cell arteritis is a medical emergency requiring prompt corticosteroid therapy to prevent visual loss. However, treatment may be associated with adverse cardiovascular effects. Atrial fibrillation is a recognized risk factor for non-arteritic ischemic optic neuropathy, creating significant diagnostic challenge when visual loss occurs in patients being treated for GCA. We present a case of a 63-year-old man initially diagnosed with GCA despite an inconclusive biopsy, who subsequently developed AF and AION. The absence of an inflammatory response and later negative multimodal investigations ultimately refuted the diagnosis of GCA, highlighting the importance of establishing diagnostic certainty to avoid harm

**Case description:**

A 61-year-old man presented to the Emergency Department in late December 2024 with severe left-sided facial pain and tenderness of the left temporal region. He denied visual symptoms, constitutional or polymyalgia rheumatica symptoms. Although a dental abscess was initially considered, subsequent assessment excluded a dental cause. Clinical examination demonstrated preserved temporal artery pulsations but significant tenderness over the left temple. Ophthalmological examination was unremarkable. Laboratory investigations revealed a C-reactive protein level of 220 mg/L. A provisional diagnosis of giant cell arteritis (GCA) was made, and treatment with prednisolone 60 mg daily was commenced. A temporal artery biopsy was performed 14 days later; however, it was non-diagnostic due to an inadequate sample. The patient experienced rapid symptomatic improvement, and inflammatory markers normalized within four weeks.In March 2025, he was admitted with new-onset atrial fibrillation and a left atrial thrombus. He was referred to a tertiary cardiac centre for catheter ablation and commenced on beta-blockers, anticoagulation, and amiodarone. During this admission, obstructive sleep apnoea was diagnosed, thought to be related to steroid-associated weight gain.

Despite persistently normal inflammatory markers, the patient reported intermittent blurring of vision in the right eye. Repeated ophthalmological assessments failed to identify a cause, and corticosteroid tapering continued. In August 2025, he developed bilateral visual loss. Ophthalmological examination demonstrated a pale right optic disc and a hyperaemic left optic disc with retinal haemorrhages. Although inflammatory markers remained normal, prednisolone was increased to 60 mg daily while urgent investigations were undertaken. Temporal artery ultrasonography was normal, and extensive investigations excluded infectious and alternative causes of anterior ischaemic optic neuropathy. Corticosteroids were rapidly withdrawn, and subsequent positron emission tomography (PET) imaging showed no evidence of large-vessel vasculitis.The patient subsequently underwent successful catheter ablation. At follow-up, visual symptoms had stabilised without further progression, although significant loss of visual acuity persisted.

**Discussion:**

This case illustrates the considerable diagnostic uncertainty that can arise in the management of giant cell arteritis. The patient’s initial presentation, characterised by temporal pain, tenderness, markedly elevated inflammatory markers, and a rapid response to corticosteroid therapy, was highly suggestive of GCA. However, despite extensive investigation, definitive confirmation of the diagnosis was never achieved. The temporal artery biopsy was non-diagnostic, while subsequent temporal artery ultrasonography and positron emission tomography failed to demonstrate evidence of cranial or large-vessel vasculitis.

This case highlights the importance of timely access to diagnostic modalities. In smaller district general hospitals, access to vascular ultrasound or advanced imaging may be limited, often necessitating empirical treatment decisions. While prompt corticosteroid therapy remains essential, diagnostic uncertainty can expose patients to prolonged treatment and significant adverse effects. In retrospect, it remains unclear whether the original diagnosis was correct and whether some of the subsequent complications could have been avoided through earlier diagnostic confirmation or exclusion of vasculitis.

The patient developed several recognised complications of long-term corticosteroid therapy, including diabetes mellitus, weight gain, and obstructive sleep apnoea. Furthermore, he subsequently developed atrial fibrillation with a left atrial thrombus. Corticosteroids are recognised to have pro-arrhythmogenic effects, and observational studies have demonstrated an increased risk of new-onset atrial fibrillation among patients receiving high-dose corticosteroid therapy.

The cause of the patient’s visual loss also remains uncertain. Although arteritic anterior ischaemic optic neuropathy (AION) was considered, alternative explanations exist. Visual symptoms occurring during corticosteroid treatment may result from cataract formation, glaucoma, transient osmotic lens changes, or medication-related toxicity. In this case, amiodarone exposure and the presence of atrial fibrillation are particularly relevant, as both have been associated with optic neuropathy and ocular ischaemic events. This case emphasises the need for multidisciplinary assessment and careful reconsideration of the diagnosis when the clinical course is atypical.

**Key learning points:**

• Giant cell arteritis may present atypically. Severe temporal pain and markedly elevated inflammatory markers may occur in the absence of visual symptoms, constitutional manifestations, or polymyalgia rheumatica, making diagnosis challenging.

• Temporal artery biopsy has important limitations. A non-diagnostic biopsy, particularly when no arterial tissue is obtained, does not exclude GCA and may necessitate reliance on clinical judgement and complementary imaging modalities.

• A rapid response to corticosteroids is supportive but not diagnostic of GCA. Clinical improvement and normalization of inflammatory markers can occur in several inflammatory conditions and should not be considered definitive evidence of vasculitis.

• Although normal inflammatory markers do not completely exclude disease activity, they have highest negative predictive value. Although ESR and CRP are valuable monitoring tools, they should be interpreted alongside.

• Visual loss in patients with suspected GCA requires urgent reassessment and consideration of alternative diagnoses. Not all ocular complications occurring during corticosteroid treatment are attributable to active vasculitis.

• Cardioembolic disease should be considered in the differential diagnosis of ischaemic optic neuropathy. The presence of atrial fibrillation and left atrial thrombus in this patient provided a plausible alternative explanation for ocular ischaemia.

• Multimodal assessment is essential. Ophthalmological evaluation, vascular imaging, cardiac investigations, and exclusion of infectious or other inflammatory causes are critical when the diagnosis remains uncertain.

• Long-term corticosteroid therapy carries significant morbidity. Steroid-associated weight gain and newly diagnosed obstructive sleep apnoea in this patient highlight the importance of balancing treatment benefits against adverse effects.

• Diagnostic uncertainty is common in vasculitis practice. Establishing mutimodal diagnostic approach in large vessel vasculitis is important to avoid unnecessary steroid exposure. This case underscores the need for ongoing reassessment of the original diagnosis when new clinical features emerge or investigations fail to support active disease.

